# The Science of Selecting Excipients for Dermal Self-Emulsifying Drug Delivery Systems

**DOI:** 10.3390/pharmaceutics15041293

**Published:** 2023-04-20

**Authors:** Daniélle van Staden, Richard K. Haynes, Joe M. Viljoen

**Affiliations:** Faculty of Health Sciences, Centre of Excellence for Pharmaceutical Sciences (Pharmacen^TM^), Building G16, North-West University, 11 Hoffman Street, Potchefstroom 2520, North-West Province, South Africa; dvanstaden711@gmail.com (D.v.S.); haynes@ust.hk (R.K.H.)

**Keywords:** excipient selection, innovative delivery systems, self-double-emulsifying drug delivery system, self-emulsifying drug delivery system, skin penetration enhancer, surfactant, topical drug delivery systems

## Abstract

Self-emulsification is considered a formulation technique that has proven capacity to improve oral drug delivery of poorly soluble drugs by advancing both solubility and bioavailability. The capacity of these formulations to produce emulsions after moderate agitation and dilution by means of water phase addition provides a simplified method to improve delivery of lipophilic drugs, where prolonged drug dissolution in the aqueous environment of the gastro-intestinal (GI) tract is known as the rate-limiting step rendering decreased drug absorption. Additionally, spontaneous emulsification has been reported as an innovative topical drug delivery system that enables successful crossing of mucus membranes as well as skin. The ease of formulation generated by the spontaneous emulsification technique itself is intriguing due to the simplified production procedure and unlimited upscaling possibilities. However, spontaneous emulsification depends solely on selecting excipients that complement each other in order to create a vehicle aimed at optimizing drug delivery. If excipients are not compatible or unable to spontaneously transpire into emulsions once exposed to mild agitation, no self-emulsification will be achieved. Therefore, the generalized view of excipients as inert bystanders facilitating delivery of an active compound cannot be accepted when selecting excipients needed to produce self-emulsifying drug delivery systems (SEDDSs). Hence, this review describes the excipients needed to generate dermal SEDDSs as well as self-double-emulsifying drug delivery systems (SDEDDSs); how to consider combinations that complement the incorporated drug(s); and an overview of using natural excipients as thickening agents and skin penetration enhancers.

## 1. Introduction

Self-emulsification is considered a formulation technique that has proven capacity to improve oral drug delivery of poorly water-soluble drugs by advancing both the solubility and bioavailability of these active compounds [1]. The capacity of self-emulsifying drug delivery systems (SEDDSs) to produce emulsions after exposure to moderate agitation and dilution by means of water phase addition provides a simplified method to improve delivery of lipophilic drugs, where prolonged drug dissolution in the aqueous environment of the gastro-intestinal (GI) tract is known as the rate-limiting step, influencing drug absorption [1,2]. Additionally, spontaneous emulsification has furthermore been reported as an innovative topical drug delivery system that enables successful crossing of mucus membranes as well as skin [3,4,5].

The ease of formulation generated by the spontaneous emulsification technique itself is intriguing due to the simplified production procedure and unlimited upscaling possibilities [1,6]. However, spontaneous emulsification depends solely on selecting excipients that complement each other in order to create a vehicle aimed at optimizing drug delivery [1,5,6,7]. If excipients are not compatible or able to trigger the spontaneous emulsification process once exposed to mild agitation, no self-emulsification will be achieved. Therefore, the generalized view of excipients as inert bystanders facilitating delivery of an active compound cannot be accepted when selecting excipients needed to produce self-emulsifying drug delivery systems (SEDDSs) [1,8]. Hence, this review describes the excipients needed to generate more specifically dermal SEDDSs as well as self-double-emulsifying drug delivery systems (SDEDDSs), how to consider combinations that complement the incorporated drug(s), and an overview of using natural excipients as potential preservatives and skin penetration enhancers.

It has been established that self-emulsification depends on the chosen lipid/surfactant combination, surfactant concentration, as well as the ratio of lipid to surfactant [9]. Findings have also confirmed that only extremely precise excipient combinations can lead to the formation of spontaneous emulsions [1,9,10]. Hence, after selecting drug(s) considered for the incorporation into SEDDSs or SDEDDSs, the screening of excipients is significant in terms of compatibility, solubility, and stability [1,9]. However, when systems with multiple excipients are designed, the evaluated excipients should produce overall solubilization instead of only exhibiting sufficient solubility of a single excipient that forms part of the formulation [1]. Therefore, the included surfactant, co-surfactant, and oil phase(s) can be of a synthetic, semi-synthetic, or natural origin as long as toxicity and tissue irritancy are considered during excipient selection [1]. Importantly, excipients are generally selected for oral SEDDSs based on their (1) capacity to attain maximum drug loading; (2) ability to ensure maximum drug absorption as influenced by sufficient duration of self-emulsification, together with minimized droplet size; (3) limiting fluctuation of droplet size influenced by the pH of the aqueous medium as well as electrolyte concentration; and (4) limiting degradation of the administered drugs in the physiological environment [1,2,9]. On the other hand, excipients selected for dermal drug delivery must be considered based on their (1) capacity to attain maximum drug loading, but to a lesser degree when compared to drugs destined for oral absorption since dermal application allows delivery of higher drug concentrations directly to the affected site; (2) ability to ensure maximum dermal drug diffusion by including excipients that facilitate diffusion by limiting trans epidermal water loss (TEWL), through hydration (water), and/or humectant inclusion; (3) limiting droplet size fluctuation and maintain a decreased droplet size to improve dermal drug delivery as achieved by selecting surface active agents capable of providing sufficient stabilization without reaching toxic surfactant concentrations; and (4) including excipients such as skin penetration enhancers in order to achieve dermal lipid disruption required to facilitate drug diffusion into underlying skin layers [5,11,12,13,14].

Considering the numerous factors mentioned, when selecting excipients for dermal SEDDSs compared to oral SEDDSs, one can also regard these same factors if aiming to create dermal SDEDDs instead of SEDDSs. Dermal SDEDDSs provide the option of including even more active ingredients in combination as these multiple emulsions can offer more options for solubilization of numerous drugs in a single formulation compared to SEDDSs that are traditionally limited to a water and oil phase supported by the surfactant phase [15]. It has been reported, for example, that incorporating quercetin into an SDEDDS improved skin permeation compared to other dermal drug delivery systems [11]. Moreover, literature concluded that a rutin-loaded non-aqueous SDEDDS exhibited a sustained release profile of up to 12 h [12]. Therefore, studies have clearly indicated the advantages of utilizing SDEDDSs as multiple-emulsion dermal drug delivery systems if approached correctly. However, only a limited literature about dermal SEDDSs and SDEDDSs has already been published, which contributes to the lack of information when aiming to select excipients during the development of these unique systems. Table 1 lists recent publications related to the successful formulation of dermal spontaneous emulsions.

Considering the summary provided in the table, it can be concluded that spontaneous emulsification is a valuable tool that should be investigated and optimized to the fullest. Therefore, this current work aims to equip the reader with an improved understanding of excipients needed to generate both dermal SEDDSs and SDEDDSs as well as the refinement of these drug delivery systems by discussing the inclusion of excipients such as thickening agents, emollients, humectants, preservatives, antioxidants, and co-solvents [5,12,16,17,18,19,20].

## 2. Components of Dermal SEDDSs/SDEDDSs

### 2.1. Drug(s) Inclusion

**Figure 1:**
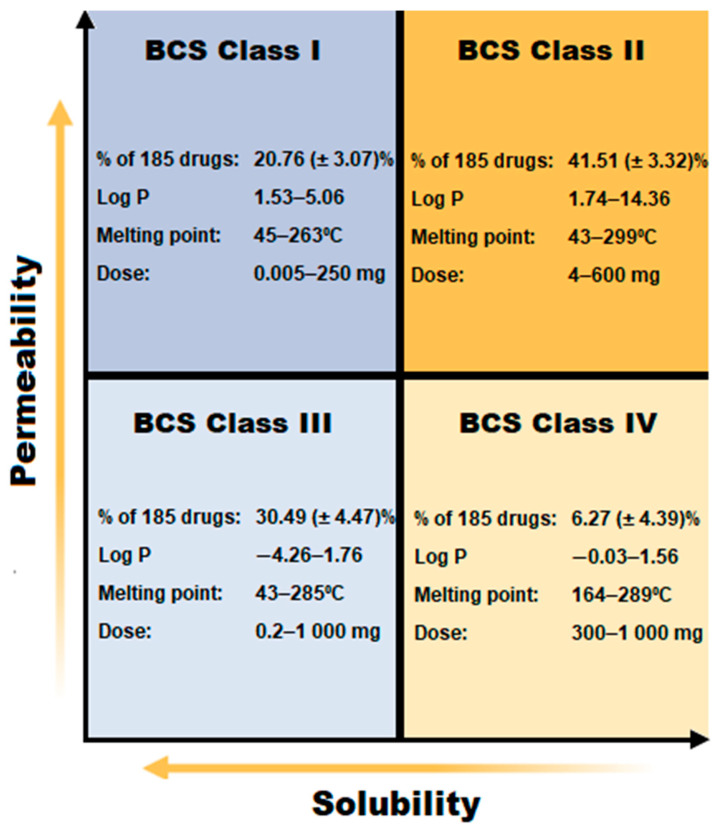
Biopharmaceutical Classification System (BCS) [23].

According to the literature, drugs considered most suitable for oral delivery via SEDDSs should preferably have a log *p* value ≥ 2, include a modest drug dosage to achieve therapeutic efficacy, and not be subjected to substantial first-pass metabolism [1,10]. If inclusion of high drug dosages is considered, remarkable solubility in at least one of the SEDDS phases should be demonstrated [1,21]. Generally, optimizing therapeutic effectiveness of orally administered SEDDSs is achieved by considering the most significant factors such as drug solubility, dissolution rate, and permeability [1]. Dissolution rate is a contributing factor that can essentially alter drug release kinetics as well as drug GI absorption [1]. These factors are best explained by the well-known Biopharmaceutical Classification System (BCS) used to predict drug solubility in the aqueous environment of the GI tract and, on the other hand, the lipophilic features of a drug that allow the crossing of membrane barriers [22]. A summary of the BCS utilized to classify drugs according to their solubility and permeability features is displayed in Figure 1.

**Figure 2:**
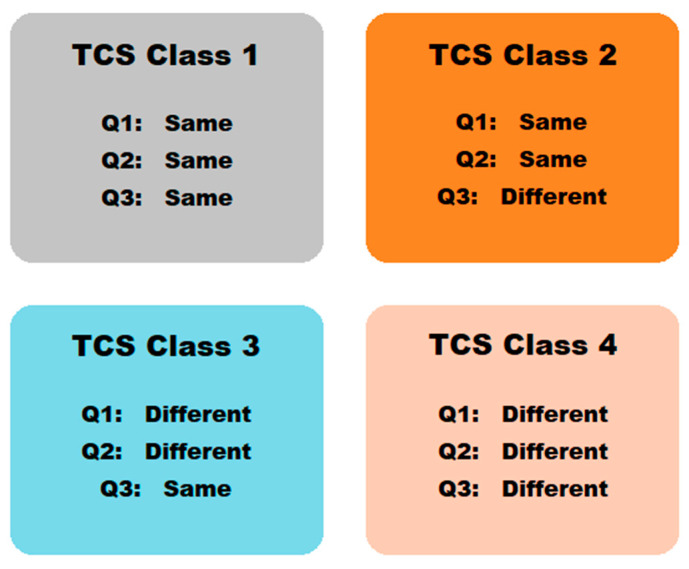
Topical Drug Classification System (TCS) [22].

Similar to the BCS employed during pre-formulation studies of oral dosage form development, the Topical Drug Classification System (TCS) has also been reported in the literature [22,24]. In the United States of America (USA), most generic topical products have qualitatively (Q1) and quantitatively (Q2) similar ingredients compared to the reference listed drug (RLD) [22,24]. The application of in vitro release (IVR) and in vitro characterization is considered for a topical dosage form of differing dosage strengths, such as suspensions, ointments, creams, and gels. Therefore, the TCS is based on the consideration of Q1, Q2, and the arrangement of matter and the microstructure of topical formulations (Q3) [22,24]. In the following, four distinct drug classes are identified by the TCS that provide pre-formulation evidence which indicates if a biowaiver can be granted for generics of topical products based on various potential scenarios that might arise during formulation development [22,24]. This system is depicted in Figure 2.

However, the TCS is limited compared to the BCS when aiming to overview a drug in terms of suitability for topical and transdermal drug delivery. Moreover, according to the authors’ knowledge, no clear classification system exists to evaluate drugs when considering drug incorporation into topical SEDDSs or SDEDDSs during the pre-formulation phase. As a lipid-based drug delivery system, SEDDSs are regarded as favorable vehicles for BCS Class II and IV drugs due to the reduced aqueous solubility of these compounds [1]. However, the decreased permeability capacity of BCS Class IV drugs creates an opportunity to put dermal SEDDSs to the test as the drug delivery vehicle must compensate for the poor permeability properties of the BCS Class IV drugs in order to facilitate dermal drug delivery. Hence, this review will provide a formulation recommendation system (FRS) in the discussion section for drugs considered for spontaneous emulsion incorporation by providing guidelines for creating a SEDDS vehicle that can improve dermal drug delivery of each BCS drug class and provide the option of including more than one drug class into a single SEDDSs or SDEDDSs. In order to present a useful and scientific FRS for SEDDSs and SDEDDSs, general excipients utilized to produce spontaneous emulsions will now be discussed.

### 2.2. Oil Phase(s)

As the lipophilic component of SEDDSs, the oil phase is crucial for solubilizing lipophilic drugs in order to enable drug delivery from either oral or dermal vehicles [14]. Improved drug solubility allows quick availability of a drug(s) for gastric absorption via the lymphatic system [1]. In terms of dermal drug delivery, enhanced solubility of a drug(s) in the oil phase presents an opportunity to introduce supersaturated SEDDSs to the skin to enhance the dermal flux of the active ingredient(s) [5]. However, one must be reminded that the release of a drug from emulsions is regulated by the polarity of the presented lipid matrix [1,13]. The polarity of any surface-active agent or lipid component is influenced by the chain length and the degree of saturation of the fatty acid composition in the lipid constituent, together with the molecular weight that impacts droplet size and size distribution, and therefore, its capacity to inhibit crystallization and sustain a supersaturated state for a longer period of time [1,25]. Moreover, characteristics such as melting point and the hydrophilic–lipophilic balance (HLB) of oils can be linked to the presence of different glycerides [1]. These glyceride features are determined with regard to glycerol content employed to generate mono-, di- or triglycerides dependent on the degree of esterification and the type of fatty acids [1]. Interestingly, medium-chain triglycerides (MCTs), classified as the presence of 6 to 12 carbon chains, render delivery of drugs into the systemic circulation via portal blood during oral administration [1]; whereas, the inclusion of long-chain triglycerides (LCTs), exceeding 12 carbon chains, leads to lymphatic system transport when utilizing oral SEDDSs [1,9]. However, oil phases comprising mainly MCTs such as ethyl oleate and caprylic/capric triglycerides are generally selected above oil phases consisting of LCTs due to improved solubility of the included drug(s), enhanced fluidity, and the improved ability to withstand oxidation reactions [1,9]. Moreover, MCTs generally improve the ease of self-emulsification compared to LCTs as these longer triglyceride chains are of a more hydrophobic nature that impede ease of emulsification [26]. Hence, MCTs will generally be favored above LCTs during the development of dermal SEDDSs as the ease of spontaneous emulsification is imperative when creating these systems. However, lymphatic drug uptake from dermal SEDDSs cannot be excluded when only employing MCTs as oil components may have access to the lymphatic system after skin application, but this differs from oral administration [5,14].

**Figure 3:**
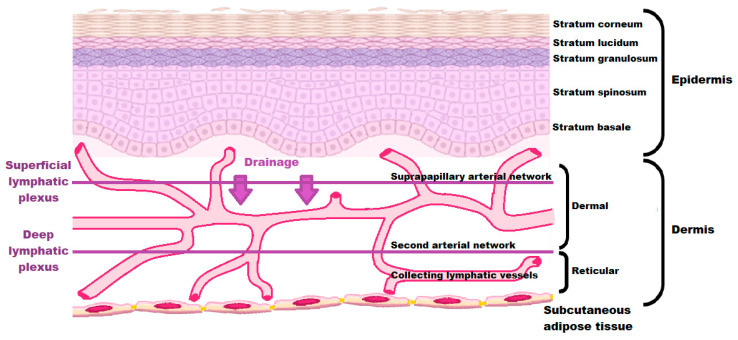
Structure of the lymphatic network of the skin.

In the skin, lymphatic vessels configure two networks as part of the one-way lymphatic system [27]. One configuration extends into the dermal papillae at a superficial level known as the superficial lymphatic plexus, whereas the second configuration is found near the subpapillary arterial network, i.e., deep lymphatic plexus [27]. The dermal lymphatic network is initially observed as capillaries of a small, blind-ended nature accompanied by minimal basement membranes that are directly linked to the extracellular matrix [27]. This link is established by anchoring filaments that allow a rapid body response to local changes observed for intestinal fluid pressure due to swelling of the extracellular matrix as brought about by pulling on filaments due to the presence of edemas that loosen the buttonlike inter-endothelial junctions [27]. Next, the described lymphatic capillaries drain into collecting vessels identified by the presence of a continuous basement membrane, coverage of smooth muscle, and a valve system that inhibit retrograde fluid flow [27]. The discussed dermal lymphatic system may be observed in Figure 3.

As stated, the routes of uptake of MCTs and LCTs from the lymphatic system, accessed by oral administration, differ from dermal applications as these lipid components must travel via the GI tract directly via portal blood to the systemic circulation, as is primarily observed with MCTs. LCTs, on the other hand, are transported via intestinal lymphatics where lipoproteins named chylomicrons, solely produced in the GI tract, transport LCTs to the lymphatic network [9]. Therefore, no clear evidence of the need to use LCTs to gain lymphatic access, in the case of dermal application, can be provided and further investigation is needed. Hence, the priority of generating SEDDSs with intensified ease of self-emulsification is considered more important than dermal lymphatic uptake. However, dermal lymphatic uptake cannot be overruled completely due to the lack of evidence.

When developing SEDDSs or SDEDDSs for dermal drug delivery, one must bear in mind the importance of selecting an oil phase with skin penetration enhancement properties. This is of substantial importance as the outermost skin layer, i.e., the stratum corneum (SC), forms a protective external barrier against toxins, pathogens, and drug molecules [28]. The SC comprises keratin-rich cells inserted in multiple lipid bilayers [28]. Therefore, skin penetration enhancers must exhibit lipid disruption properties to gain drug entry into the underlying skin layers to achieve therapeutic effects. Lipid disruption can be attained by active or passive means [28,29]. Active lipid disruption refers to the employment of devices, i.e., microneedles, thermal ablation, micro-dermabrasion, electroporation, and cavitational ultrasound, that are used effectively but are restricted to utilization on small skin areas as use on larger skin areas may be difficult [28]. Passive lipid disruption, alternatively, is achieved by chemical permeation enhancers that affect lipid disruption through lipid extraction, fluidization, or introducing other mechanisms of structural reorganization [28]. However, a balance must be maintained between achieving desired lipid-disruptive properties and avoiding dermal irritation due to lipid disruption [28]. Chemical penetration enhancer examples include azone derivates, alcohols, esters, glycols, fatty acids, pyrrolidones, surfactants, terpenes, and sulphoxides [28]. These compounds are deemed small molecules (<500 Da) that can penetrate the skin in increased quantities, which can lead to skin irritation, cytotoxicity, or irreversible skin barrier alteration [28]. Therefore, focus has been shifting towards utilizing skin penetration enhancers obtained from a more natural origin [5]. For instance, olive oil is a rich source of oleic acid and can be included as a natural oily source of this well-known skin penetration enhancing fatty acid that provides dermal lipid disruption without irritation [5,13,30]. Interestingly, the extent of glyceride saturation is considered more important in terms of topical/transdermal drug delivery compared to oral drug delivery, as numerous publications have confirmed that unsaturated fatty acids (UFAs) have superior dermal lipid-disruptive properties compared to saturated fatty acids (SFAs) [14,31,32]. This discovery can be linked to the reduced capacity of SFAs to dissolve within the natural lipids of the SC, which renders diminished lipid disruption accompanied by less effective skin penetration enhancement [14,31,32]. Additionally, during the selection of the oil phase in terms of dermal drug delivery, one must consider the sensation experienced by patients when applying the dosage form onto their skin.

It has been reported that stickiness and greasiness of dosage forms such as ointments decrease patient compliance [33]. Hence, the skin sensation provided by the oil as well as the organoleptic properties of the oil should also be contemplated. For instance, sweet almond oil is referred to in the literature as a lightweight oil accompanied by a mild smell [34]. However, although this oil may possess acceptable organoleptic properties, oils retrieved from nut origin should be used with caution due to the possibility of patients being allergic to nuts [14]. On the other hand, jojoba oil renders an excellent lubricity without greasiness, as is the case with other lipids such as lanolin and petrolatum [35]. Rice bran oil is described as a “natural and value-added healthy ingredient” in terms of enhancing skin moisture and contributing towards improved physical stability of emulsions [36]. Thus, the lipid component of SEDDSs or SDEDDSs can influence patient compliance. Yet, in terms of dermal drug delivery, the challenge remains to select a lipid component with sufficient cutaneous lipid-disruptive properties while also limiting TEWL to assist with dermal drug diffusion by means of occlusivity without leaving behind a sticky and unpleasant skin sensation.

Then again, when considering different oil phases as potential lipid components of SDEDDSs compared to SEDDSs, selection is even further complicated when having to ensure that oils included into SDEDDSs exhibit immiscibility in order to establish an internal and external oil phase when developing water-in-oil-in-oil (w/o/o) emulsions as well as oil-in-oil-in-water (o/o/w) emulsions [37]. Traditional (aqueous) SDEDDSs provide the advantageous capacity to incorporate hydrophilic drugs together with decreased concentrations of hydrophobic drugs as two water phases are present to dissolve water-soluble drugs [37]. However, the development of non-aqueous SDEDDSs, where two oil phases and a single water phase are present, can provide similar advantages compared to traditional SDEDDSs, apart from increasing the concentration of the fat-soluble drug that can be incorporated [37]. Hence, internal oil phases can be described as polar organic solvents combined with non-polar natural oils, utilized as the external oil phase, to maintain distinct phase separation [37]. Moreover, the inclusion of a polar internal oil phase establishes increased drug solubilization achieved by increased drug solubility observed in the internal oil phase compared to the external oil phase [37]. Additionally, this creates the opportunity to select an internal oil phase that optimizes drug solubility while including an external oil phase with favorable but less desirable solubility properties that optimizes skin penetration enhancement and ensures maximized dermal drug delivery. However, to stabilize these unique multiple emulsions, often large concentrations of surfactants are employed that can disturb physiological skin processes upon chronic application [37].

### 2.3. Surfactant(s) and Co-Surfactant(s)

Surfactants can induce phospholipid emulsification, which leads to cellular damage that results in a cytolytic process accompanied by the release of proteins, lysosomal and cytoplasmic enzymes, as well as inflammatory mediators [38]. Consequently, phospholipid emulsification normally establishes skin irritation, especially if a patient is frequently exposed to increased surfactant concentrations during topical application of topical/transdermal treatment. However, surfactants are essential ingredients included in SEDDSs as well as SDEDDSs for the purpose of stabilizing emulsions. Therefore, a scientific balance must be maintained between including sufficient surfactant concentrations required for stabilization while keeping concentrations low enough to avoid potential skin irritation upon chronic exposure during prolonged treatment periods. A potential solution is to use both a surfactant and a co-surfactant since the literature states that the inclusion of a co-surfactant can simulate similar stabilizing effects compared to increasing the concentration of an individual surfactant [1,39]. This can decrease the amount of individual surfactant needed by approximately 30% [1,39]. Another option is to use non-ionic surfactants during the development of dermal drug delivery vehicles, as these compounds tend to establish moderate, irreversible changes to membranes while being non-toxic [1,14].

The main objective for surfactant inclusion is to decrease interfacial tension by forming an interfacial film that allows dispersion formation [1,14], whereas co-surfactants decrease the transitory negative value of the established interfacial tension even further [1]. This provides flexibility to the interfacial film that enables formation of varied curvatures for creation of different microemulsion concentrations [1]. The contact enlargement results in the scattering of fine droplets [1]. Next, more surfactant or a higher ratio of surfactant to co-surfactant will be absorbed until the film is depleted enough to maintain positive interfacial tension that results in spontaneous emulsification. Co-surfactants generally comprise medium-chain length alcohols (C3–C8) such as ethanol, isopropyl alcohol, 1-butanol, and propylene glycol [1,40].

**Figure 4:**
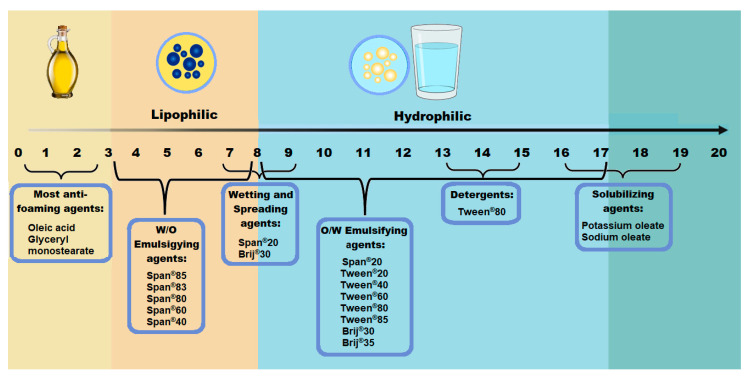
Hydrophilic–Lipophilic Balance (HLB) Scale [42,43].

The most established tool utilized during emulsion formulation is calculating the hydrophilic–lipophilic balance (HLB) values of surfactants and co-surfactants utilized in combination prior to formulation [1]. This tool, though not perfect, is employed to determine the ratio of surfactant to co-surfactant as well as consider different surface-active agent combinations if a fixed ratio is chosen in advance. Fixed ratios of surfactant to co-surfactant can be selected for SEDDSs and SDEDDSs in advance as the literature has confirmed the formation of more stable SEDDSs at a ratio of 1:1 (surfactant:co-surfactant), whereas decreased stability causes precipitation of the incorporated drug despite an enlarged emulsion range [5,41]. Generally, surfactants with an HLB value exceeding 12 are selected to achieve improved emulsification of SEDDSs [1]. The HLB scale together with examples of HLB values of different excipients can be seen in Figure 4.

As illustrated in Figure 4, traditionally the HLB scale ranges from 1 to 20 despite reporting HLB values that exceed 20 [44]. An HLB value ranging from 1 to 10 signifies a more lipophilic compound [44,45], whereas an HLB value between 11 and 20 indicates a more hydrophilic nature [44,45,46]. This is influenced by the number of hydrophilic groups present in each molecule. Hence, combinations of lipophilic and hydrophilic surface-active agents can generate an optimized HLB value [44,45,46]. In this case, an optimized HLB value refers to choosing between formulating an oil-in-water (o/w) emulsion or a water-in-oil (w/o) emulsion.

When considering dermal drug delivery, the choice between an o/w or w/o emulsion can be influenced by numerous factors. Firstly, o/w emulsions are more susceptible to oxidation reactions [47]. This increased oxidative susceptibility is linked to a higher extent of interfacial interactions provided by the lipid component and prooxidants such as metal ions in the aqueous phase due to the greater surface area provided by oil droplets [47]. The literature suggests that lipid oxidation observed in w/o emulsions should occur at a rate similar to that of bulk oils since the surface of the oil phase is directly exposed to air [47]. Consequently, the need to discuss and investigate the inclusion of excipients such as antioxidants during formulation of SEDDSs and SDEDDSs will be addressed in another section of this review.

Next, the nature of the incorporated drug(s) can influence the type of emulsion. This statement is based on the principle that a drug should be dissolved in the emulsion to diffuse across the SC into the underlying skin layers [5,14,15]. Therefore, if a lipid-soluble drug is incorporated, it will mainly be dissolved within the lipid phase of an emulsion [48]. The SC is known for its dominant lipid arrangement [49]. Thus, a lipid-soluble drug can benefit from inclusion into an o/w emulsion as the drug would desire escape from the predominantly aqueous environment and diffuse into the lipophilic SC when made possible by the inclusion of skin penetration enhancers and the hydrating effect contributed to water inclusion [5,14,49,50,51]. However, w/o emulsions are notorious for improved occlusive effects that limit TEWL and prolong contact time between the formulation and the skin that in turn improves dermal drug diffusion [5,14,51]. Therefore, considering these few mentioned factors, a one-size-fits-all approach is insufficient during development of topical/transdermal SEDDSs and SDEDDSs as careful consideration must be given to the nature of the drug(s) as well as the stability of the formulations during selection of surface-active agents. The selection of an intended HLB value prior to formulation is beneficial as it can decrease the number of pre-formulation experiments during development of SEDDSs and SDEDDSs destined for dermal drug delivery.

Finally, the HLB values considered for multiple emulsions such as SDEDDSs will differ as definite inclusion of both a lipophilic and a hydrophilic surface-active agent is needed to establish formulation stability at the different interfaces present within multiple emulsions [52]. For this reason, during development of multiple emulsions, one surface-active agent must provide stabilization of the primary emulsion and a second surface-active agent should provide stabilization of the secondary emulsion formation [52].

### 2.4. The Water Phase

The final essential component for uncomplicated SEDDSs and SDEDDSs to form spontaneously is the hydrophilic component. Water can be described as the primary skin penetration enhancer as its presence on the skin establishes hydration that leads to loosening of the tightly organized lipid structure of the SC [14,49,53]. Moreover, water is essential in providing an aqueous medium needed to dissolve hydrophilic drugs incorporated into SEDDSs or SDEDDSs. The necessity of including water in spontaneous emulsions destined for dermal application can be considered the most significant difference between oral spontaneous emulsions and dermal spontaneous emulsions [14]. Normally, an oily concentrate together with a surfactant phase is administered orally; and only after administration is it introduced to the aqueous environment of the GI tract, where, once inside the body, spontaneous emulsification can occur [1]. However, contrary to this type of delivery system, spontaneous emulsification of dermal SEDDSs and SDEDDSs must occur prior to application as sweat secretion is erratic and unpredictable. It can therefore not be regarded as the sole water component for spontaneous emulsification upon dermal application due to high inter-patient variability as well as the influence that the dermal condition being treated may have on the delivery system (e.g., secretions) [54,55]. Importantly, different dermal diseases can alter skin structure, either temporarily or permanently, due to factors such as skin hydration, climate effects, and the causative organism [54,55]. Therefore, water inclusion or inclusion of a polar solvent is considered imperative during formulation of dermal spontaneous emulsions.

## 3. The Addition of Auxiliary Excipients

### 3.1. Why the Need for Refinement of Topical/Transdermal Spontaneous Emulsions?

After reviewing the main components utilized for the preparation of spontaneous emulsions, it is time to consider the effect of auxiliary additives that can potentially benefit dermal drug delivery as well as emulsion stability. Dermal permeation of drugs can be influenced by the type of emulsion prepared with reference to o/w and w/o emulsions as well as different types of multiple emulsions [56]. Generally, drug(s) dissolved in the external continuous phase of an emulsion should exhibit permeation behavior that differs from drug(s) incorporated into the internal dispersed phase droplets [56]. For instance, droplet size of the internal phase should notably influence dermal permeation [56]. However, the addition of auxiliary excipients during the formulation of both SEDDSs and SDEDDSs can assist in creating stable as well as elegant emulsions while also considering modification of dermal drug delivery [56]. For example, the inclusion of a thickening agent can aid in the formation of a more occlusive, uniform, and stable emulsion [56].

### 3.2. Some Examples of Auxiliary Excipients That Can Be Included into SEDDSs and SDEDDSs for Topical/Transdermal Drug Delivery

#### 3.2.1. Thickening Agents

The addition of a thickening agent directly impacts the viscosity of a formulation [56,57,58]. Nevertheless, the literature is still debating whether the thickening of formulations does in fact hinder or rather enhance dermal permeation of drugs [56,57,58]. It has for example been reported that the inclusion of a thickening agent, i.e., xanthan gum, improved dermal drug permeation due to increased stability and homogeneity of the said emulsion [56]. This can be attributed to a significant reduction in droplet size as drugs dissolved in internal phase droplets were able to diffuse with increased success over the obstacle provided by the SC, specifically due to the decreased droplet size. The smaller droplet size enlarges the surface area of the oil droplets, which in turn increases the contact area between the oil droplets and the skin, inducing higher skin permeation of the incorporated drugs. Moreover, it was found that the increased viscosity established occlusivity that prolonged the time the skin was exposed to the formulation, which consequently facilitated higher dermal drug permeation [56]. Additionally, this decreased droplet size also exhibited increased emulsion stability [56]. Contrarily, another study established that increased viscosity could restrict diffusion of a drug through the formulation, impeding dermal drug diffusion [58]. Furthermore, another study aimed at delivering berberine from chitosan hydrogel bases concluded that diffusion of berberine through the gel-like base into the aqueous external phase was restricted due to the increased viscosity of the formulation [57]. However, the same study found that increased doses of incorporated berberine rendered enhanced dermal drug delivery despite the use of high-viscosity formulations [57]. Moreover, the inclusion of skin penetration enhancers, in this case the non-ionic surfactant Tween^®^80, was emphasized to enable drug release from formulations of high viscosity [57]. Lastly, hydrophobic bentonite clay was employed together with non-ionic surfactants to assist with co-stabilization of non-aqueous SDEDDSs [16]. Hence, thickening agents can assist with dermal drug delivery from SEDDSs and SDEDDSs that might be of a low viscosity but easily transpire into emulsions.

#### 3.2.2. Emollients and Humectants

Emollients are added to formulations for the purpose of improving spreadability, improving skin feel, as well as improving the hydration function of the SC [59,60,61]. The softening of skin brought about by emollients is attributed to the formation of an occlusive film on the skin that renders a reduced TEWL [62]. They are nonetheless mostly considered cosmetic ingredients instead of pharmaceutical ingredients [60]. Consequently, emollients are included in lotions, creams, and ointments to adjust consistency of cosmetic vehicles for the purpose of providing a softer and smoother skin feel [63]. However, the contribution of emollients with regards to dermal drug delivery should not be underestimated. Emollients do not only enhance the character of the applied vehicle but also promote dermal penetration of incorporated drug(s) [63]. Moreover, emollients have demonstrated the capacity to reduce stress experienced by the SC, such as injuries or wounds, by penetrating the SC and replacing the lost water volume [61]. This emphasizes the profound relationship between the transport properties provided by emollients and their beneficial effect on repairing the barrier function of the outermost skin layer [61].

A model was suggested by Berkey et al. [61] to consider both the penetration volume of emollients, i.e., drug transport potential in the SC, as well as the biomechanical stress reduction on the SC upon emollient application. This model can greatly assist during evaluation of emollients for inclusion into spontaneous emulsions. Their study concluded the importance of reviewing viscosity, molecular mass, and diffusivity [61]. Simply stated, small molecules generally demonstrate increased diffusivity, which leads to improved dermal penetration [61]. However, the emollient glycerol is discussed as an example where a decreased molecular mass rendered increased diffusivity as expected but not exceptional SC penetration volume values [61]. This is attributed to the more viscous nature of glycerol as well as its higher water solubility compared to other emollients tested during this study [61]. In addition, topological surface area (TPSA) consideration can also provide valuable information with regards to the determination of penetration volume as it contributes towards understanding emollient bonding interactions with SC components [61]. By taking TPSA into consideration, together with molecule size and polarity, it was concluded that smaller molecules of low polarity tend to demonstrate improved penetration of the SC lipid matrix [61]. Interestingly, this signifies the importance of not exclusively using log *p* values of emollients as a penetration prediction measurement since log *p* increases while polarity is reduced and decreases as the molecular weight is reduced [61].

Cosmetic emollients such as dipropyl heptyl carbonate, dibutyl adipate, and propyl heptyl caprylate are often confused with dermatological emollients, which refer to lotions, creams, and ointments utilized to improve skin barrier function during eczema treatment [61]. Similarly, the mechanisms of emollients and humectants are easily confused during formulation of topical preparations. Emollients establish reduced TEWL by generating an occlusive film on the skin that fills cracks and injuries to repair SC barrier function, whereas humectants draw moisture from the environment and lock the attracted moisture in the skin to improve dermal hydration [61,64]. Humectants are useful in countering the drying effect that surfactants tend to have on the skin [65]. Humectants are frequently paired with occlusive agents such as petrolatum to provide protection against drying skin in the winter months when less moisture is available for humectants to lock inside the SC [66]. Examples of humectants include hyaluronic acid, urea, glycerol, and propylene glycol [64,67]. Some excipients, such as glycerol, can be used as either an emollient or humectant based on the concentration of glycerol included in a formulation [68].

#### 3.2.3. Preservatives and Antioxidants

The addition of a hydrophilic component in SEDDSs and SDEDDSs raises the need for preservative inclusion for the purpose of inhibiting microbial growth [69]. Additionally, the presence of a lipophilic phase signifies the importance of including an antioxidant since lipids tend to spontaneously participate in autoxidation with atmospheric oxygen [70]. Moreover, depending on the preservative antioxidant function, photoprotective properties can also be provided [69]. However, the undeniable global concern raised by the toxicity, sensitization, and irritation of preservatives cannot be ignored [69,71]. For example, the preservative triclosan may lead to the disruption of sexual hormones as well as the deregulation of the thyroid function [59,69,72]. Furthermore, the famously phenyl-, benzyl-, pentyl-, isopropyl-, and isobutyl parabens have been banned from cosmetic products by the European Commission (EC)-amended Annexures II and V of the EU Cosmetic Regulation since 2014 due to no submitted safety evaluation data [69,71,73]. Therefore, preservatives mark an ethical area of formulation where safety should be considered above the functionality of ingredients [69]. Fortunately, similar to the employment of natural oils as lipid components, in order to avoid skin irritation and make use of natural skin penetration enhancers, preservatives from a natural origin are widely and successfully researched. An excellent example is with regards to the use of *Nigella sativa* L. seed oil as a natural preservative [74,75], which is already used in the food industry as a natural preservative of Mediterranean cheeses [75]. Another example includes cinnamaldehyde, which is an active monomer isolated from the bark of cinnamon stems and used as a preservative in both the cosmetic and food industries [76]. Likewise, excipients utilized during formulation of mucolytic SEDDSs, such as conventional cationic surfactants, can potentially be used as multi-purpose ingredients if a proper risk-benefit assessment is made [3]. Therefore, inclusion of preservatives into topical/transdermal SEDDSs and SDEDDSs can be beneficial to prolong the shelf-life of these spontaneous emulsions.

#### 3.2.4. Co-Solvents

Co-solvents are frequently included during the formulation of both mucoadhesive and solidified SEDDSs intended for oral drug delivery [77,78,79]. Therefore, the possibility as well as benefits of utilizing co-solvents during the preparation of dermal SEDDSs and SDEDDSs should be reviewed. This can be of particular importance during the delivery of lipophilic drugs that should be presented in higher concentrations to achieve therapeutic effects. Moreover, inclusion of co-solvents such as ethanol in dermal formulations can lead to the evaporation of the co-solvent during application, which can render an increased flux of drug across the SC due to maintained supersaturated drug concentrations [80]. The choice of solvent can also greatly benefit skin penetration enhancement if it is a solvent known for SC lipid-disruptive properties [80]. Evaporative systems provide non-occlusive, passive delivery of drugs without the disadvantage of causing skin irritation [80]. Moreover, these are quick drying systems which improve patient compliance [80]. However, supersaturated formulations are known as inherent thermodynamically unstable systems, which in turn may cause drug crystallization [80]. Fortunately, drug crystallization can be prevented by including antinucleating polymers that either inhibit nucleus formation or impede crystallization [80].

Instead of evaluating the addition of a co-solvent, one can rather consider developing SDEDDSs instead of SEDDSs. This statement is made after reflecting on the literature regarding the development of topical/transdermal SDEDDSs by creating non-aqueous multiple emulsions produced by spontaneous emulsification [12,37]. The main aims during formulation of SDEDDSs are to improve the solubility of the incorporated drug(s), use the lowest surfactant concentration possible, and improve skin retention time compared to other dermal vehicles [37]. Hence, the addition of a third emulsion phase can assist in improving solubility while decreasing surfactant concentrations and improving skin retention time. Solubility can be increased by selecting an oil phase with a polar nature such as Transcutol^®^ or glycerol [12,37]. Moreover, surfactant phase concentrations can be limited to a minimum amount if pre-formulation studies are performed by evaluating oil phase immiscibility and determining the self-emulsification capacity of SDEDDSs by constructing pseudo-ternary phase diagrams [5]. Lastly, skin retention time can be improved due to the possibility of utilizing an external oil phase capable of excellent SC lipid disruption to facilitate enhanced dermal drug delivery [15]. In addition, multiple emulsions, such as SDEDDSs, provide the opportunity to achieve sustained release during delivery of both an individual drug as well as more than one drug, since drugs of different solubilities will favor certain phases of the emulsions and remain in these solvents at concentrated quantities compared to other phases [15,81]. For this reason, sustained release can occur according to diffusivity of the inner phases across the outermost phase prior to reaching the SC.

## 4. Discussion

**Figure 5:**
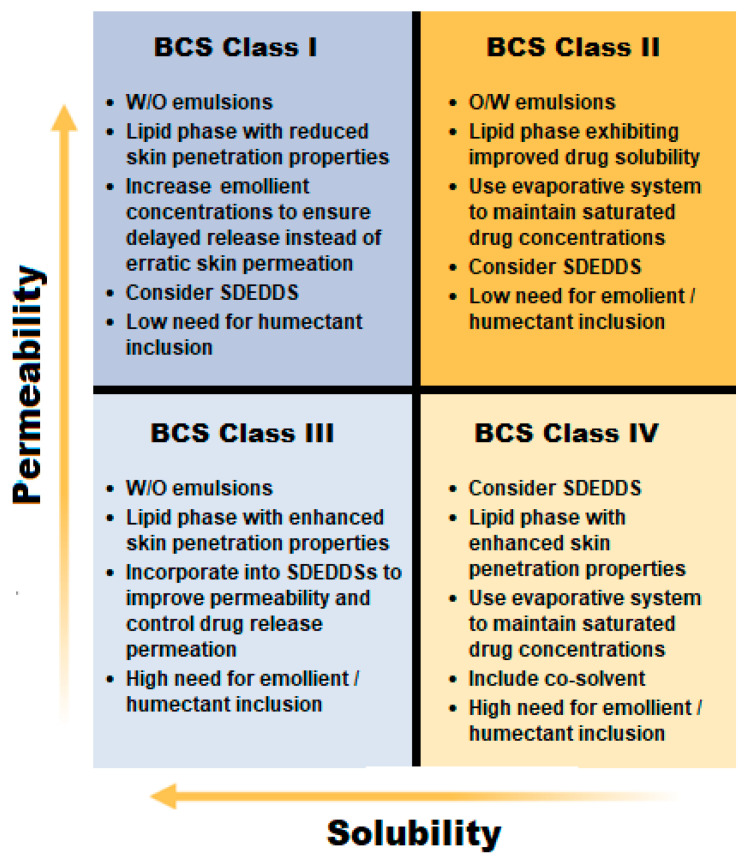
Formulation recommendation system (FRS) when considering drugs belonging to different biopharmaceutical classifications for incorporation into dermal spontaneous emulsions.

This paper provides insight into recent innovations in terms of dermal spontaneous emulsions compared to oral spontaneous emulsions. The capacity of these formulations to cross barriers such as mucus membranes and the skin provide evidence of their versatility if designed correctly. However, as seen by the detailed description of general excipients as well as auxiliary compounds, the generalized view of excipients as inert bystanders cannot be accepted when aiming to develop dermal SEDDSs or SDEDDSs. Self-emulsification depends solely on the selected lipid/surfactant combination, surfactant concentration, as well as the ratio of lipid to surfactant. Therefore, extremely precise excipient combinations are essential to achieve formation of spontaneous emulsions. Consequently, after selecting drug(s) that are to be included into spontaneously formed emulsions, excipient screening and selection are important in terms of compatibility, solubility, and stability. Furthermore, excipients selected for SEDDSs and SDEDDSs should (1) have the capacity to attain maximum drug loading, but to a lesser extent compared to oral SEDDSs as dermal application of drugs allow delivery of higher drug concentrations directly to the affected area; (2) optimize dermal diffusion by selecting excipients that facilitate transport due to minimized TEWL by means of hydration and/or humectant inclusion; (3) ensure uniform droplet size distribution and maintain reduced droplet sizes for the purpose of improving diffusivity across the SC; and (4) utilize skin penetration enhancers in order to ensure SC lipid disruption that leads to drug diffusion into underlying skin layers. With the purpose of optimizing the development of dermal SEDDSs and SDEDDSs, this review provides an FRS that offers guidelines in terms of excipient selection for different BCS drug classes as displayed in Figure 5.

As demonstrated in Figure 1, BCS Class I drugs have high permeability and high aqueous solubility [23]. Therefore, this review recommends attempting delivery of these types of drugs from w/o emulsions, as the external lipid phase should provide sufficient skin penetration enhancement properties while the drug is mainly concentrated in the internal water phase. If a BCS Class I drug exhibits excellent permeability across the SC, the external oil phase can be selected to restrict diffusion, which in turn will render controlled drug release during dermal application. This restricted diffusion is also of importance when aiming to deliver a BCS Class I drug into the skin layers (topically) instead of establishing systemic drug delivery (transdermal). Lipids comprising elevated SFA levels can be contemplated as a good choice, as SFAs are known to cause decreased lipid disruption compared to UFAs [14,31,32]. On the other hand, emollient inclusion will influence viscosity and may establish delayed drug release compared to formulations of lower viscosity [57,58]. Moreover, utilizing SDEDDSs instead of SEDDSs can furthermore provide delayed as well as controlled drug release of BCS Class I drugs, especially if therapy with more than one drug is desired. In addition, BCS Class I drug delivery will not necessarily benefit highly from humectant inclusion since these drugs are known to have high permeability properties and humectant inclusion improves dermal drug permeation.

Next, BCS Class II drugs can be recommended for inclusion in either w/o or o/w emulsions. This statement is made bearing in mind the benefits of delivering a lipophilic drug from a w/o system where the external oil phase provides a larger medium to dissolve the drug into compared to o/w emulsions, while also providing skin penetration enhancement due to the presence of skin penetration enhancers in the oil phase. However, it can be reasoned that a lipophilic drug will remain trapped in a mainly lipophilic vehicle instead of crossing the lipophilic SC [82,83], hence risking a reservoir effect in the SC without diffusivity into underlying skin layers. Therefore, despite the advantage of utilizing increased oil concentrations to fully dissolve BCS Class II drugs, an oil phase should be included that renders increased drug solubility in lower concentrations while incorporating BCS Class II drugs into o/w emulsions. Likewise, co-solvent inclusion as well as generating SDEDDSs instead of SEDDSs can contribute toward increased drug solubility, which leads to enhanced dermal drug delivery [80]. Furthermore, SDEDDSs provide the possibility of optimizing drug solubility while controlling dermal drug permeation, as BCS Class II drugs are limited in terms of aqueous solubility and not drug permeability [84]. Last, evaporative drug delivery systems can similarly be considered in order to maintain saturated drug concentrations at the skin surface to benefit dermal diffusion of a BCS Class II drug [80].

On the other hand, BCS Class III drugs will be more suitable for w/o emulsion inclusion as these drugs have sufficient aqueous solubility while needing assistance with membrane permeation [23]. Therefore, an oil phase with high dermal lipid-disruptive properties should be included [32]. Moreover, a SDEDDS formulation will benefit these drugs as a multiple-emulsion formulation provides the alternative of including powerful skin penetration enhancers in different phases that can establish controlled skin permeation [15]. Emollient or humectant inclusion will greatly benefit BCS Class III drugs since these auxiliary excipients improve dermal drug permeation by limiting TEWL [62,66].

Finally, BCS Class IV drugs are problematic due to both poor permeability and aqueous solubility [23]. However, dermal drug delivery is not considered impossible if the drug delivery vehicle is designed correctly. The inclusion of potent skin penetration enhancers are vital in order to establish SC lipid disruption for the purpose of achieving dermal drug permeation [32]. Alternatively, utilizing an evaporative drug delivery system to improve drug solubility by maintaining saturated drug concentrations at the skin surface can lead to increased dermal flux [80]. Furthermore, co-solvent inclusion and/or SDEDDS development should receive serious consideration [15]. SDEDDSs are of particular advantage during delivery of BCS Class IV drugs, as non-aqueous SDEDDSs can be utilized to optimize drug solubility while achieving effective dermal drug diffusion due to the reversible SC lipid disruption made possible through the inclusion of natural skin penetration enhancers [5,14,32]. In addition, emollient or humectant inclusion should not be forgotten as these excipients, as stated previously, can contribute toward enhanced dermal drug diffusion by limiting TEWL while creating drug delivery channels for drug diffusion between the strict structure of the SC lipids [58]. If BCS Class IV drugs are compared to BCS Class II drugs, it can be reasoned that either w/o or o/w emulsion inclusion will suffice if effective skin penetration enhancers and drug solubilizers are incorporated. However, multiple emulsions such as SDEDDSs seem to be a more promising alternative as both o/w and w/o emulsions are restricted in terms of excipient quantity and compatibility if desiring spontaneous emulsification together with enhanced skin penetration and improved drug solubility. Therefore, SDEDDSs can provide an alternative dosage form to optimize BCS Class IV dermal drug delivery due to their capacity to include more excipients to assist with improved drug delivery.

## 5. Conclusions

As spontaneous emulsions develop as a dosage form able to cross barriers such as mucus membranes and skin, the importance of developing spontaneous multiple emulsions should not be ignored. Formulation of multiple emulsions is considered complex as o/w and w/o systems must exist in a single system [84]. However, these systems are known to be of a finer texture and smooth touch upon application [84]. Moreover, multiple emulsions can protect drugs from degradation and establish controlled drug release rates [84]. This controlled release can be established by the capacity of multiple emulsions to create internal depots by entrapping drugs from an external diluted continuous phase [84]. Furthermore, double emulsions are also able to improve solubility of drugs [84]. Hence, the above can provide a solution to delivering both drugs of poor permeability and those with increased lipophilicity, which are known to impede successful dermal drug delivery.

This review aimed to provide insight into the main excipients of dermal spontaneous emulsions, such as the lipid, water, and surfactant components, as well as preparation methods of SEDDSs. Additionally, the potential role of auxiliary excipients during formulation of dermal spontaneous emulsions was also discussed. Emphasis must be placed on the importance of spontaneous emulsification and excipient compatibility between the main components of spontaneous emulsions; and that auxiliary excipients are not responsible for establishing spontaneous emulsification but instead are included to provide extended emulsion shelf-life while improving dermal drug delivery. Moreover, future prospects of dermal spontaneous emulsions can include the investigation of different formulation techniques and the effect that these different techniques may have on factors such as droplet size and ease of dermal diffusion. Therefore, to the knowledge of the authors, this is the first review of its kind suggesting refinement of self-emulsified drug delivery systems destined for dermal drug delivery by including auxiliary excipients well known to the pharmaceutical industry.

## Figures and Tables

**Table 1:** Recent developments published on dermal spontaneous emulsions.

Dosage Form	Year of Publication	Drug	BCS ^1^ Drug Class	Reference
SDEDDS	2015	Nattokinase	Enzyme	[16]
SNEDDS ^2^	2018	Curcumin	Class IV	[17]
SEDDS	2020	Clofazimine	Class II	[5]
SEDDS	2020	Rose bengal	Xanthene	[18]
N-SDEDDS ^3^	2021	Rutin	Class II	[12]
Nanoemulgel (prepared from SEDDS)	2021	Chrysin	Class II	[19]
SNEDDS ^2^	2023	Astaxanthin	Class IV	[20]

^1^ Biopharmaceutical classification system; ^2^ self-nano-emulsifying drug delivery system; ^3^ non-aqueous self-double-emulsifying drug delivery system.

## Data Availability

Not applicable.
